# *Notes from the Field:* COVID-19 Pandemic-Related Changes in Blood Lead Screening — Chicago, Illinois, 2017–2022

**DOI:** 10.15585/mmwr.mm7250a4

**Published:** 2023-12-15

**Authors:** Hillary Spencer, Emile Jorgensen, Jennifer Seo, Candice Robinson

**Affiliations:** ^1^Epidemic Intelligence Service, CDC; ^2^Chicago Department of Public Health, Chicago, Illinois.

Lead is an environmental hazard that can cause serious harm to young children. Early childhood lead exposure can damage the brain and nervous system, slow growth and development, and cause hearing and speech problems (*1*). Screening for elevated blood lead levels (BLL) is essential for routine care of young children. At the onset of the COVID-19 pandemic, substantial disruptions to health care access occurred for routine, preventive care (*2*). At the onset of the pandemic (January–May 2020), 34% fewer U.S. children aged <6 years received blood lead level testing than during the same months in 2019 (*3*). The Chicago Department of Public Health (CDPH) characterized patterns of blood lead testing among young children in Chicago from the onset of the pandemic in 2019 through 2022.

## Investigation and Outcomes

All children residing in Chicago should be screened for their BLL at age 12, 24, and 36 months (*4*). Illinois requires that all BLL results be reported to the state surveillance system; data are shared with CDPH.* The current analysis included all BLL test results from Chicago residents aged 11–48 months, to include recommended screening ages and a makeup period given potential delayed screening at the onset of the pandemic. March–September was selected as the comparison period to account for observed seasonality of lead testing,^†^ effects of the stay-at-home mandate, and the largest disruptions to health care during the first year of the pandemic. The total number of lead tests performed during March–September was divided by the American Community Survey 5-year population estimate of children aged <5 years.^§^ The average during 2017–2019 was assigned as the prepandemic baseline. The effect was evaluated geographically by assigning community areas (77 static administrative units were defined by the city for long-term statistical and tracking purposes^¶^) as low, medium, or high risk for BLLs exceeding the Blood Lead Reference Value on the basis of historic lead levels (average rank during 2010–2018).Within each of the three risk categories, the total decrease was calculated as well as the median decrease of the community areas making up each risk category (from 2019 to 2022**). This activity was reviewed by CDC, deemed not research, and was conducted consistent with applicable federal law and CDC policy^.^^††^

The ratio of BLL tests performed among Chicago children aged 11–48 months per 100 children aged <5 years in 2020 (14.7) was 29.1% lower than the annual average during 2017–2019 (20.7) (Figure). The ratio increased during 2021 (17.5) and 2022 (18.5) but remained 15.4% and 10.6% lower than during the 2017–2019 baseline, respectively. The prepandemic testing ratio was highest among the 25 community areas with the highest risk for BLLs exceeding the Blood Lead Reference Value; the decrease in testing was also greater in these highest risk areas (total decrease = 18%; median decrease = 17.7%) than in the 25 at medium risk (total = 10.1%; median = 7.6%) and the 24 at low risk (total = 8.8%; median = 7.7%).

**FIGURE:**
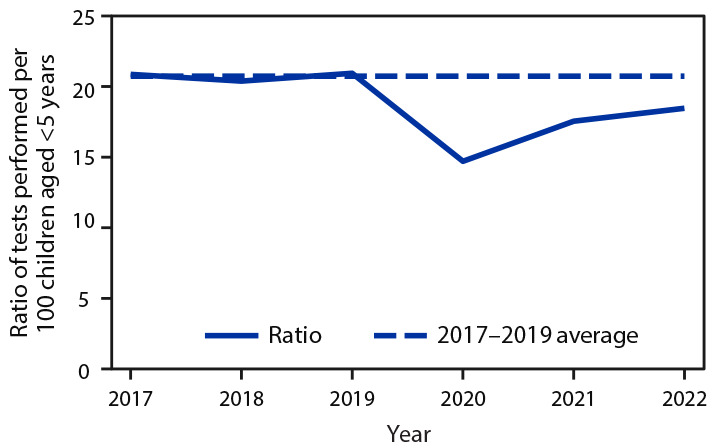
Blood lead level testing* among children aged 11–48 months — Chicago, Illinois, 2017–2022 * During March–September.

## Preliminary Conclusions and Analysis

BLL testing in young children decreased at the pandemic onset in 2020. Testing increased in 2021 and again in 2022 but did not return to prepandemic baseline in Chicago. Community areas with the highest average rank of BLLs exceeding the Blood Lead Reference Value during 2010–2018 were most affected by pandemic-related decreases in testing. Pandemic-related changes in blood lead testing might have exacerbated longstanding health inequities. The most affected community areas should receive increased attention to promote BLL testing among children at the highest risk for lead exposure. It is important for jurisdictions to evaluate pandemic-related changes in blood lead testing for young children and consider including geographic or demographic unit analyses to assess impact on the most vulnerable communities.

## Footnotes

*
https://dph.illinois.gov/content/dam/soi/en/web/idph/files/77-iac-845.pdf

† In typical years, an increase in lead tests is observed in late summer and a decrease in early winter, likely reflecting the school-year cycle because blood lead testing is required for entry into child care facilities, preschools, and kindergarten.

§ The 2022 American Community Survey was not yet available. The citywide population of children aged <5 years in 2022 was extrapolated from the preceding years (2017–2021) to account for the change in population.

¶ Three community areas were excluded from this analysis: two because of a limited population and one because of historical reporting errors.

** The population denominator for community areas used was the 2021 5-year estimate (2017–2021), because the population change is not uniform across community areas.

†† 45 C.F.R. part 46, 21 C.F.R. part 56; 42 U.S.C. Sect. 241(d); 5 U.S.C. Sect. 552a; 44 U.S.C. Sect. 3501 et seq.

## References

[1] CDC. Health effects of lead exposure. Atlanta, GA: US Department of Health and Human Services, CDC; 2022. Accessed January 6, 2023. https://www.cdc.gov/nceh/lead/prevention/health-effects.htm

[2] Lebrun-Harris LA, Sappenfield OR, Warren MD. Missed and delayed preventive health care visits among US children due to the COVID-19 pandemic. Public Health Rep 2022;137:336–43. 10.1177/00333549211061322 34969335 PMC8900224

[3] Courtney JG, Chuke SO, Dyke K, Decreases in young children who received blood lead level testing during COVID-19—34 jurisdictions, January–May 2020. MMWR Morb Mortal Wkly Rep 2021;70:155–61. 10.15585/mmwr.mm7005a2 33539334 PMC7861485

[4] Illinois Department of Public Health Lead Program. Childhood blood lead evaluation and testing recommendations. Springfield, IL: Illinois Department of Public Health Lead Program; 2019. https://dph.illinois.gov/content/dam/soi/en/web/idph/files/forms/childleadevaluationtestingrecommendations.pdf

